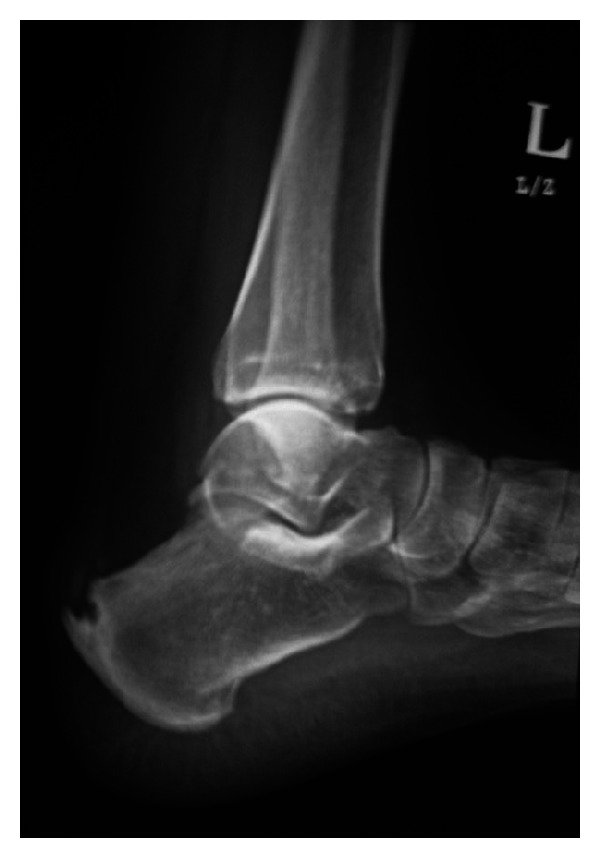# Could Ossification of the Achilles Tendon Have a Hereditary Component?

**DOI:** 10.1155/2013/539740

**Published:** 2013-04-30

**Authors:** Chawki Cortbaoui, Jihad Matta, Rayan Elkattah

**Affiliations:** ^1^Department of Orthopedic Surgery and Traumatology, Saint Georges University Medical Center, Balamand University, P.O. Box 166378, Achrafieh, Beirut 1100 2807, Lebanon; ^2^Department of Diagnostic Radiology, American University of Beirut Medical Center, P.O. Box 110236, Beirut 1107 2020, Lebanon

## Abstract

Ossification of the Achilles tendon (OTA) is an unusual clinical condition. It is characterized by the presence of an ossified mass within the fibrocartilaginous substance of the Achilles tendon. The etiology of the ossification of the Achilles tendon is unknown. Review of the literature suggests that its etiology is multifactorial. The major contributing factors are trauma and surgery with other minor causes such as systemic diseases, metabolic conditions, and infections. To our knowledge, no previous reports suggest any genetic/hereditary predisposition in OAT. We report 3 siblings who have OAT with no history of any of the aforementioned predisposing factors. Could OAT have a hereditary component as one of its etiologies?

## 1. Introduction

Ossification of the Achilles tendon (OAT) is an unusual clinical condition. Since its description in 1908, there have been sporadic reports of this condition in the medical literature [1]. It is characterized by the presence of an ossified mass within the fibrocartilaginous substance of the tendon either within the body of the tendon or at its insertion into the calcaneous and is usually a firm, tender mass [1–3] or can be completely asymptomatic. Discomfort or pain may be due to local “inflammation” or a fracture through the ossified mass that should be suspected and treated [2, 3]. Ossification occurs twice as frequently in males with no age predilection [2–4]. Extensive ossification involving greater than 50% of the tendon bulk is particularly unusual [2, 4].

The etiology of OAT is unknown [5] however, review of the literature suggests that its etiology is multi-factorial. The major contributing factors are trauma and surgery with other minor causes such as systemic diseases, metabolic conditions, and infections. To our knowledge, no previous reports suggest any genetic/hereditary predisposition in OAT. We report 3 siblings that have OAT with no history of any of the aforementioned predisposing factors. Could OAT have a hereditary component as one of its etiologies?

## 2. Case 1

**Figure 1:**
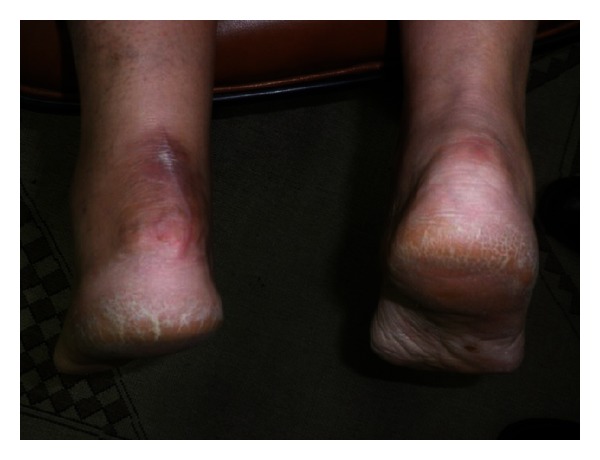


**Figure 2:**
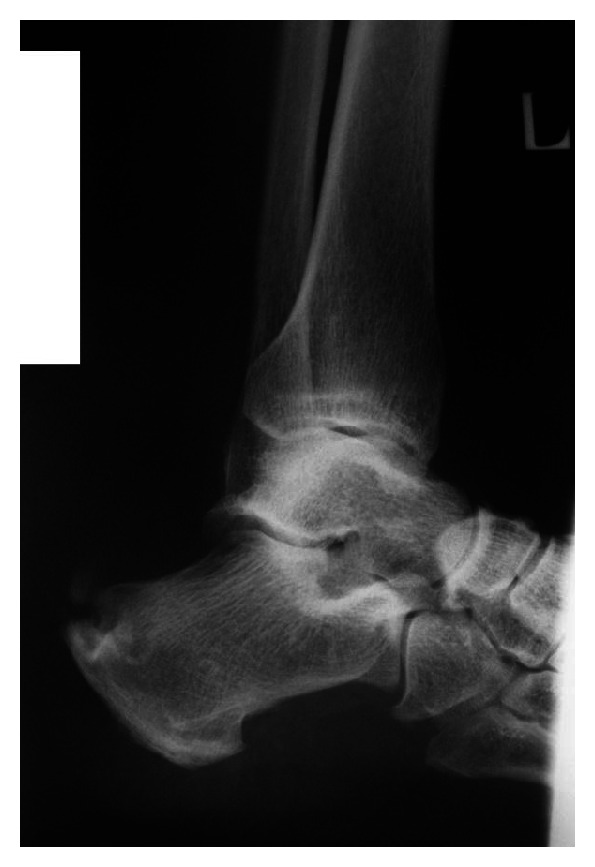


A 50 year old previously healthy female with no known systemic or metabolic illness, presented with increasing bilateral heel pain of one year duration (left more than right). Pain was exacerbated by initiation of walking and slightly relieved by analgesics. It was associated with a non-inflamed but tender bulge (Figure 1). Plain radiography of the left ankle revealed a 2 centimeter ossification within the Achilles tendon proximal to the level of its insertion into the calcanium (Figure 2). Surgical treatment was sought as conservative treatment was ineffective. Histologic analysis of the excised fragments revealed dystrophic calcification of the tendon with fragments of medullary (spongy) bone with no other pathologic findings.

## 3. Case 2

**Figure 3:**
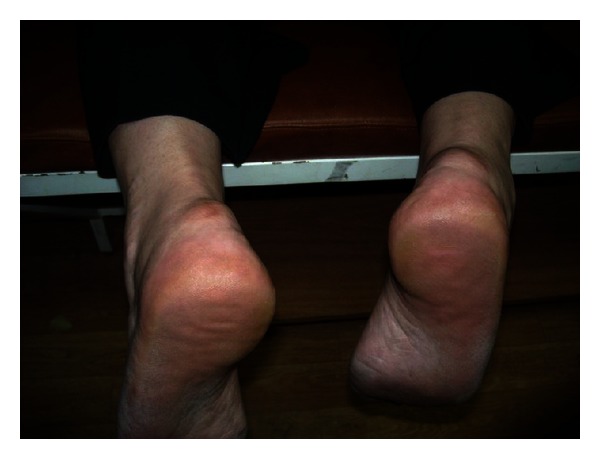


**Figure 4:**
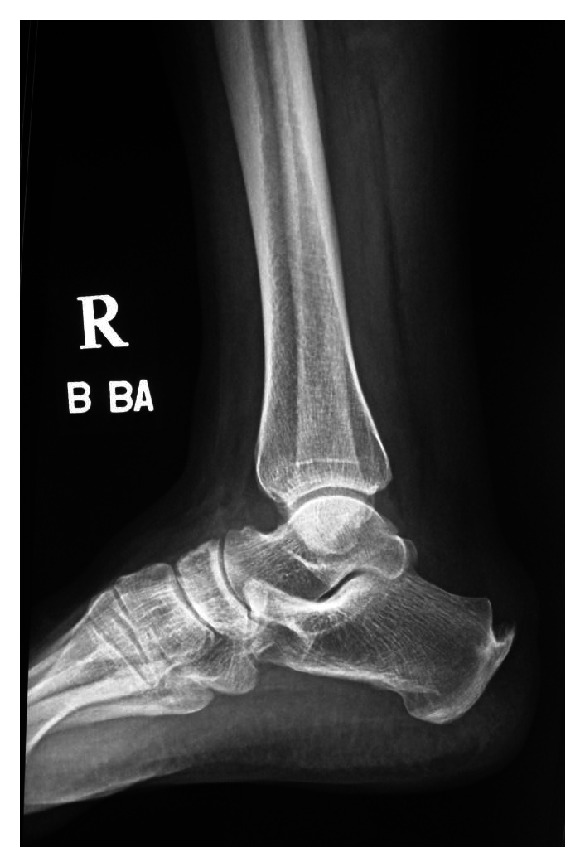


**Figure 5:**
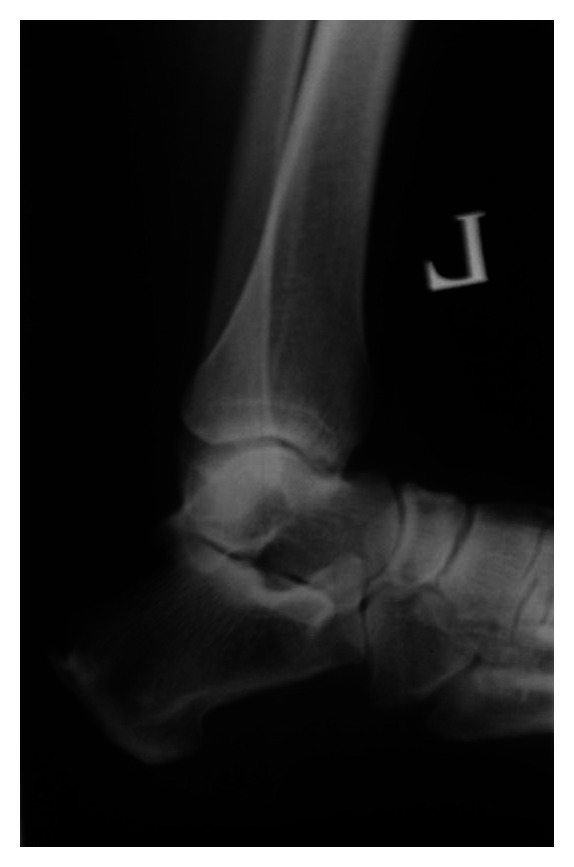


A 64 year old previously healthy female with no known systemic or metabolic illness, presented with bilateral heel pain of 6 months duration (right same as left). Pain was exacerbated by walking and was associated with a non-inflamed tender bulge (Figure 3). Spur-like ossifications (1.5 centimeter and 1 centimeter in the right and left Achilles tendons, resp.) were seen on plain radiography (Figures 4 and 5). A trial of non-steroidal anti-inflammatory drugs (NSAID) alleviated the pain. 

## 4. Case 3

**Figure 6:**
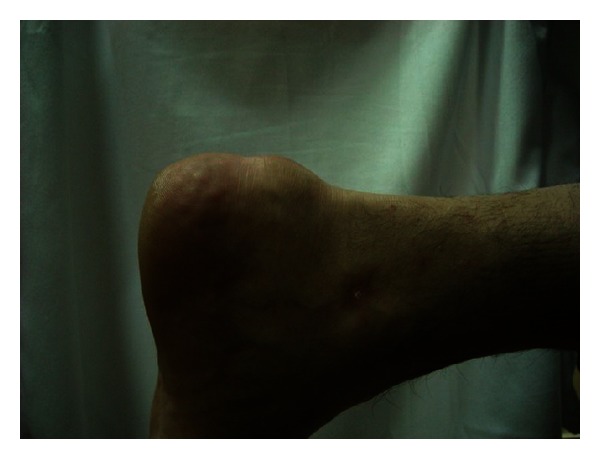


A 54 year old previously healthy male with no known metabolic or systemic illness, presented with stable non-remitting bilateral heel pain of 7 months duration (right same as left) (Figure 6). Pain was exacerbated by walking and responded well to NSAID therapy. Plain radiography of the left calcanium revealed a 1 centimeter ossification within the Achilles tendon proximal to the level of its insertion into the calcanium and a spur-like ossification at the insertion of the Achilles tendon into the calcanium (Figure 7).

*Note*. All 3 cases had no known history of recent or old trauma or surgery in the ankle area.

## 5. Discussion

OAT is a definite, yet rare, clinical entity [5]. The most commonly described etiologies are previous trauma (tendon rupture or repeated micro-trauma) and surgery (previous Achilles tendon surgery, clubfoot surgery, surgery for cerebral palsy). Patients with systemic diseases or metabolic conditions may also demonstrate OAT such as in diabetes, Wilsons disease, fluorosis, renal failure, Reiter's syndrome, ankylosing spondylitis, gout, Diffuse Idiopathic Skeletal Hyperostosis (DISH), sero-negative arthropathies, and infectious causes (syphilis, gastrocnemius abscess and osteomyelitis) [1–4, 6]. Sasaki et al. reported that calcification and ossification are probably a consequence of degenerative changes in collagen, the etiology of which may be related to vascular insufficiency [7].

OAT causes discomfort, restriction of motion, and disability of daily activities [7]. Heel pain with associated mass and swelling [8] or mass alone, is the most common presentation of OAT. Plain film radiography usually reveals a calcified mass with evidence of a fracture line or simply a calcified portion of the Achilles tendon with free bone fragments or ossifications within the body of the tendon. Conservation methods are adequate and are most commonly used. The treatment of choice is excision of the calcified mass, repair of the Achilles tendon, and plaster immobilization for at least six to eight weeks [5].

Plain film radiography reliably demonstrates the ossified area and the contour of the Achilles tendon in the majority of affected patients [1]. A classification system for OAT has been described by Morris et al. based upon the location of the ossified area on radiographs [9]. Accordingly, all three patients in our report can be classified as type 1a.

Surgery may be indicated when there is pain or fracture. Removal of the ossified portion may require reconstruction of the tendon, particularly in large areas of ossification where the large intratendinous gap makes direct repair unsuitable. Various reconstructive methods have been described with the aim of preserving the tendon [2]. 

Histologic analysis of OAT constitutes one of the following patterns; enchondral and intramembranous ossification, lamellar bone, conglomerate foci of calcification, or dystrophic calcification in connective tissue. This may be in favor of a multi-factorial etiology [2, 4].

## 6. Conclusion

All three patients in our report presented for evaluation of a painful mass/bulge proximal to the heel. They are siblings and one of them had bilateral ossifications evident on plain film radiography, and were all classified as type 1a on Morris classification. They all had a negative history of trauma, surgery, and any of the previously mentioned systemic, metabolic, and infections etiologies. However, in view of such a non-revealing history in three siblings, the possibility of a genetic predisposition was contemplated. Whether acquired or inherited, the management of OAT is the same. Future cases are needed to confirm our suspicion. Until then, “idiopathic” OAT could in fact be hereditary and this likelihood is to be kept in mind.

## Figures and Tables

**Figure 7:**